# Comparative Effectiveness of Triphala and Conventional Root Canal Irrigants in Primary Teeth: A Systematic Review

**DOI:** 10.7759/cureus.98788

**Published:** 2025-12-09

**Authors:** Pranjal Chavan, Padawe Dimple, Vilas Takate

**Affiliations:** 1 Department of Pediatric and Preventive Dentistry, Government Dental College and Hospital, Mumbai, Mumbai, IND

**Keywords:** antimicrobial efficacy, pediatric endodontics, primary teeth, root canal irrigants, triphala

## Abstract

The present systematic review aimed to evaluate the effectiveness of Triphala, a traditional Ayurvedic herbal formulation, as a root canal irrigant in primary teeth compared to conventional irrigants such as sodium hypochlorite, chlorhexidine, and saline. A comprehensive electronic search was conducted across multiple databases, including PubMed, MEDLINE, Embase, Scopus, and Cochrane Library, and supplemented by grey literature. Studies published in English between January 2000 and February 2025 were considered. Five studies met the inclusion criteria, comprising randomized controlled trials, in vitro, and ex vivo studies, all of which were conducted in India. The findings revealed that Triphala possesses significant antimicrobial activity against endodontic pathogens such as *Enterococcus faecalis* and *Candida albicans*, though sodium hypochlorite consistently showed greater microbial reduction. One study reported comparable long-term clinical and radiographic success between Triphala and chlorhexidine over a 12-month period. Variability in concentration, formulation, and methodology contributed to differences in efficacy across studies. Triphala demonstrated a favorable safety profile and biocompatibility, supporting its potential as an alternative or adjunctive irrigant in pediatric endodontics. However, current evidence is limited by geographic concentration and methodological heterogeneity. Further high-quality multicenter trials are warranted to validate its clinical applicability and promote its broader integration into pediatric dental practice.

## Introduction and background

The maintenance of primary dentition is a critical aspect of pediatric oral healthcare, serving multiple functional and developmental roles such as proper mastication, speech articulation, aesthetic harmony, and guidance for the eruption of permanent successors [1]. Premature loss of primary teeth due to carious lesions or pulpal pathologies can result in space loss, eruption disturbances, and malocclusion, thereby impacting long-term oral health [2]. Hence, effective endodontic intervention in primary teeth is essential to preserve dental integrity and support the child’s overall growth and development.

Pulpectomy, or root canal treatment in primary teeth, is the preferred modality when the radicular pulp is irreversibly inflamed or necrotic [3]. In simple terms, pulpectomy involves the removal of diseased pulp tissue, followed by thorough cleaning, shaping, disinfection, and obturation of the canal system using resorbable materials suitable for exfoliating teeth [4]. However, anatomical variations in primary teeth, such as tortuous and ribbon-shaped canals, multiple accessory canals, and large apical foramina, pose significant challenges to complete mechanical debridement [5]. Furthermore, the close proximity of primary roots to developing permanent tooth buds necessitates a cautious approach during instrumentation and irrigation to avoid iatrogenic harm [6].

Chemical irrigation serves as a vital adjunct to mechanical preparation in ensuring adequate disinfection of the root canal system [7]. Irrigants perform key roles, including microbial reduction, smear layer removal, organic tissue dissolution, and canal lubrication [8]. Ideally, an irrigant should exhibit broad-spectrum antimicrobial action, tissue dissolution capability, low surface tension, biocompatibility, and compatibility with obturating materials [9]. No single solution fulfills all these requirements, so combinations and adjunctive agents are commonly used in clinical practice.

Sodium hypochlorite (NaOCl) is widely accepted as the gold standard irrigant due to its potent antimicrobial and tissue-dissolving abilities through the release of hypochlorous acid [10]. Nevertheless, in pediatric dentistry, its high cytotoxicity, unpleasant taste, risk of chemical burns, and potential allergic reactions are significant drawbacks [11]. Chlorhexidine (CHX) gluconate offers long-lasting antimicrobial substantivity and is generally well tolerated, but lacks tissue-dissolving capacity and forms potentially toxic precipitates when mixed with NaOCl [12,13]. Ethylenediaminetetraacetic acid (EDTA) is effective in removing the inorganic component of the smear layer but exhibits limited antimicrobial activity [14]. In parallel, contemporary work on intracanal dressings and medicaments, such as comparisons between calcium hydroxide pastes and calcium hydroxide-impregnated gutta-percha points, highlights how formulation and delivery can markedly influence antimicrobial performance against resistant species like *Enterococcus faecalis* [15]. Collectively, these limitations have prompted exploration of safer, more biocompatible alternatives, particularly in children.

Among various herbal candidates, Triphala has shown promising potential in endodontics. It is a traditional Ayurvedic polyherbal formulation composed of the dried fruits of *Emblica officinalis*, *Terminalia bellirica*, and *Terminalia chebula* [16,17]. Experimental and in vitro studies have demonstrated antimicrobial, anti-inflammatory, antioxidant, and tissue-healing properties for its constituent plants [18-21]. Proposed mechanisms include disruption of bacterial cell membranes, interference with enzymatic activity, and modulation of biofilms through polyphenol-mediated chelation and free-radical scavenging [22,23]. However, these mechanisms are predominantly derived from laboratory and model-based investigations, and direct clinical confirmation in primary teeth remains limited. Importantly, Triphala has been reported to exhibit relatively low cytotoxicity to host tissues compared with many synthetic chemotherapeutic agents, which may be advantageous in the pediatric population [24].

Multiple clinical and in vitro studies have highlighted the efficacy of Triphala against endodontic pathogens such as *E. faecalis* and *Candida albicans*, with some findings suggesting parity or even superiority to NaOCl under specific experimental conditions [25-29]. Triphala has also been investigated for its potential to contribute to smear layer modification and biofilm disruption, particularly when used with activation methods [30]. Nonetheless, these data are largely laboratory-based, and translation to clinical practice in primary teeth is not straightforward. Clinical trials in pediatric endodontics are relatively few, vary in their Triphala formulations and irrigation protocols, and differ in outcome measures and follow-up periods.

Consequently, while the preclinical evidence base suggests that Triphala is a biologically active and potentially safer irrigant, pediatric dentists currently lack synthesized, clinically oriented evidence to determine whether Triphala is a viable alternative or adjunct to conventional irrigants in routine pulpectomy. In particular, there is a need to collate and critically appraise data on microbial reduction, clinical and radiographic success, and any adverse effects associated with Triphala use in primary teeth.

Therefore, this systematic review aims to comprehensively evaluate and compare the effectiveness of Triphala and conventional root canal irrigants (such as NaOCl, CHX, and EDTA) in primary teeth, with a specific focus on (i) microbial reduction, (ii) short- and long-term clinical and radiographic outcomes, and (iii) reported adverse effects. By synthesizing the available clinical and experimental evidence, this review seeks to inform evidence-based decision-making in pediatric endodontics and to clarify the potential role of Triphala as a safer, natural adjunct or alternative to established irrigation protocols.

## Review

Methodology

The present systematic review was conducted in accordance with the Preferred Reporting Items for Systematic Reviews and Meta-Analyses (PRISMA) 2020 guidelines to ensure methodological transparency and reproducibility [31]. The review protocol was registered a priori with the International Prospective Register of Systematic Reviews (PROSPERO; registration ID CRD42024622972), representing the modern era of evidence-based endodontics and the emergence of Triphala as a potential irrigant.

Review Objective

The primary objective of this systematic review was to evaluate and compare the effectiveness of Triphala, a polyherbal formulation, with conventional root canal irrigants such as NaOCl, CHX, and EDTA in the management of pulpally involved primary teeth. Outcomes of interest included microbial reduction, clinical success, radiographic healing, and the incidence of adverse effects.

Eligibility Criteria

Studies were selected based on predefined inclusion and exclusion criteria framed using the Population, Intervention, Comparison, Outcome, and Study design (PICOS) model. The review included randomized controlled trials (RCTs), controlled clinical trials (CCTs), prospective cohort studies, and in vitro/ex vivo studies involving primary teeth. For clinical studies, pediatric patients up to 12 years of age with primary teeth requiring pulpectomy due to pulpitis or necrosis were eligible. Studies were included if they used Triphala in any concentration or formulation as a root canal irrigant, either alone or as an adjunct, and compared it with conventional chemical irrigants. Outcomes assessed had to include at least one of the following: microbial reduction (quantitative microbiology or molecular methods), clinical success (pain relief, absence of infection), radiographic healing (resolution of periapical radiolucencies), or any reported adverse effects. Only studies published in English between January 2000 and February 2025 were considered to capture contemporary evidence-based endodontic practice. Studies were excluded if they involved permanent teeth or mixed dentition, used Triphala in combination with other herbal agents without separate evaluation, or were animal studies, retrospective designs, case series, case reports, or narrative reviews. No restrictions were placed on the country of origin; however, all studies that ultimately met the inclusion criteria were from India. The PICOS framework is summarized in Table 1.

**Table 1:** PICOS framework with inclusion and exclusion criteria CFU: colony-forming unit; PCR: polymerase chain reaction

PICOS element	Inclusion criteria	Exclusion criteria
Population	Pediatric patients (≤12 years) with primary teeth requiring root canal treatment due to pulpitis or necrotic pulp; both single- and multi-rooted primary teeth; extracted primary teeth used in in vitro/ex vivo studies	Studies involving permanent teeth or mixed dentition; patients with systemic conditions severely affecting oral health; case reports or studies without defined control groups
Intervention	Use of Triphala as a root canal irrigant, either as primary or adjunctive irrigation; any concentration or formulation of Triphala; studies reporting clinical and/or microbiological outcomes	Studies using Triphala in combination with other herbal agents without independent analysis; animal studies; studies not clearly describing the Triphala protocol
Comparator	Use of conventional root canal irrigants such as sodium hypochlorite (NaOCl), chlorhexidine (CHX), EDTA, or other standard irrigants	Studies without a comparator group; studies comparing Triphala with non-irrigant or non-standard interventions
Outcomes	Primary outcome: microbial reduction (e.g., CFU counts, PCR analysis). Secondary outcomes: clinical success (pain relief, absence of swelling or infection), radiographic healing, adverse effects, patient/operator preference, cost-effectiveness	Studies lacking outcome data related to antimicrobial efficacy or clinical success; studies reporting only subjective outcomes without microbiological or radiographic validation
Study Design	Randomized controlled trials (RCTs); controlled clinical trials (CCTs); prospective cohort studies; in vitro and ex vivo studies involving primary teeth	Retrospective studies; case series or case reports; narrative reviews, editorials, and letters to the editor

Information Sources and Search Strategy

A comprehensive electronic search was carried out across multiple databases, including PubMed, MEDLINE, Embase, Scopus, Cochrane Library, and Google Scholar. The electronic search covered the period from January 2000 to February 2025 and was last updated in February 2025. Additional searches were conducted in grey literature sources, including academic theses, conference abstracts, and clinical trial registries such as ClinicalTrials.gov and the WHO International Clinical Trials Registry Platform (ICTRP). The search strategy incorporated a combination of keywords and Medical Subject Headings (MeSH) terms such as “Triphala,” “root canal irrigant,” “primary teeth,” “pediatric endodontics,” “herbal irrigant,” and “microbial reduction,” with Boolean operators used to refine and expand the search. Filters were applied to include only English-language publications involving human subjects for clinical studies. Full database-specific search strings (including Boolean operators and filters) for each database are provided in the Appendices for replicability.

Study Selection Process

Study selection was conducted in two phases. In the first phase, two reviewers independently screened the titles and abstracts of retrieved studies for relevance. In the second phase, full-text articles of potentially eligible studies were retrieved and evaluated against the inclusion and exclusion criteria. Disagreements at any stage were resolved through discussion or consultation with a third reviewer.

Data Extraction

A standardized data extraction form was used to collect pertinent details from each included study. Extracted data included study identifiers (author, year, country), study design (RCT, CCT, cohort, in vitro, ex vivo), sample size and characteristics (age, gender, tooth type, clinical or laboratory setting), intervention details (Triphala formulation and concentration, irrigation protocol), comparator details (type and concentration of conventional irrigant, protocol), and outcomes (microbial reduction metrics, clinical findings, radiographic healing, adverse events, patient/operator preference, and follow-up duration, where applicable). Two reviewers independently extracted data, and discrepancies were resolved by mutual consensus or third-reviewer adjudication. Any uncertainties or missing information were noted and considered during the quality appraisal.

Risk of Bias (RoB) Assessment

The RoB in included studies was assessed using design-appropriate tools. For RCTs, the Cochrane Risk of Bias 2.0 (RoB 2) tool was applied to evaluate domains such as randomization, deviations from intended interventions, missing outcome data, outcome measurement, and selective reporting [32]. Each domain was rated, and an overall judgment (low, some concerns, or high RoB) was assigned. For in vitro and ex vivo studies, the Quality Assessment Tool for In Vitro Studies of Dental Materials (QUIN) was employed to assess factors including clarity of aims, sample size justification, randomization, blinding of outcome assessment, adequacy of control groups, and appropriateness of statistical analysis [33]. Two reviewers independently performed the risk-of-bias assessments, with disagreements resolved through discussion; formal kappa statistics were not calculated, which is acknowledged as a methodological limitation. Final risk ratings are presented in the Results section.

Data Synthesis

Given the small number of included studies and substantial heterogeneity in study design (in vitro, ex vivo, in vivo), Triphala formulations and concentrations, comparator irrigants, outcome measures (colony-forming unit (CFU) counts, turbidity, zone of inhibition, clinical and radiographic endpoints), and follow-up durations, a quantitative meta-analysis was not considered appropriate. We therefore employed a narrative synthesis. Studies were grouped by comparator irrigant (e.g., Triphala vs. NaOCl, Triphala vs. CHX, Triphala vs. saline) and by outcome domains (microbial, clinical, radiographic). Within each group, we summarized the direction and magnitude of effects, highlighting where conventional irrigants were superior, where Triphala showed comparable outcomes, and where results were inconsistent. Detailed numerical results are presented in summary tables, while the text focuses on key trends and clinically relevant comparisons.

Assessment of Quality of Evidence

The GRADE (Grading of Recommendations Assessment, Development and Evaluation) approach was used to appraise the overall certainty of evidence for each primary comparison (e.g., Triphala vs. NaOCl; Triphala vs. CHX; Triphala vs. saline) for the outcome of antimicrobial efficacy, and, where applicable, for clinical and radiographic outcomes [34]. Evidence was classified into four levels, including high, moderate, low, or very low, based on RoB, inconsistency, indirectness, imprecision, and potential publication bias. Heterogeneity in designs and outcomes, limited clinical data, and imprecision in effect estimates led to downgrading of certainty for some comparisons, particularly those involving NaOCl.

Results

**Figure 1:**
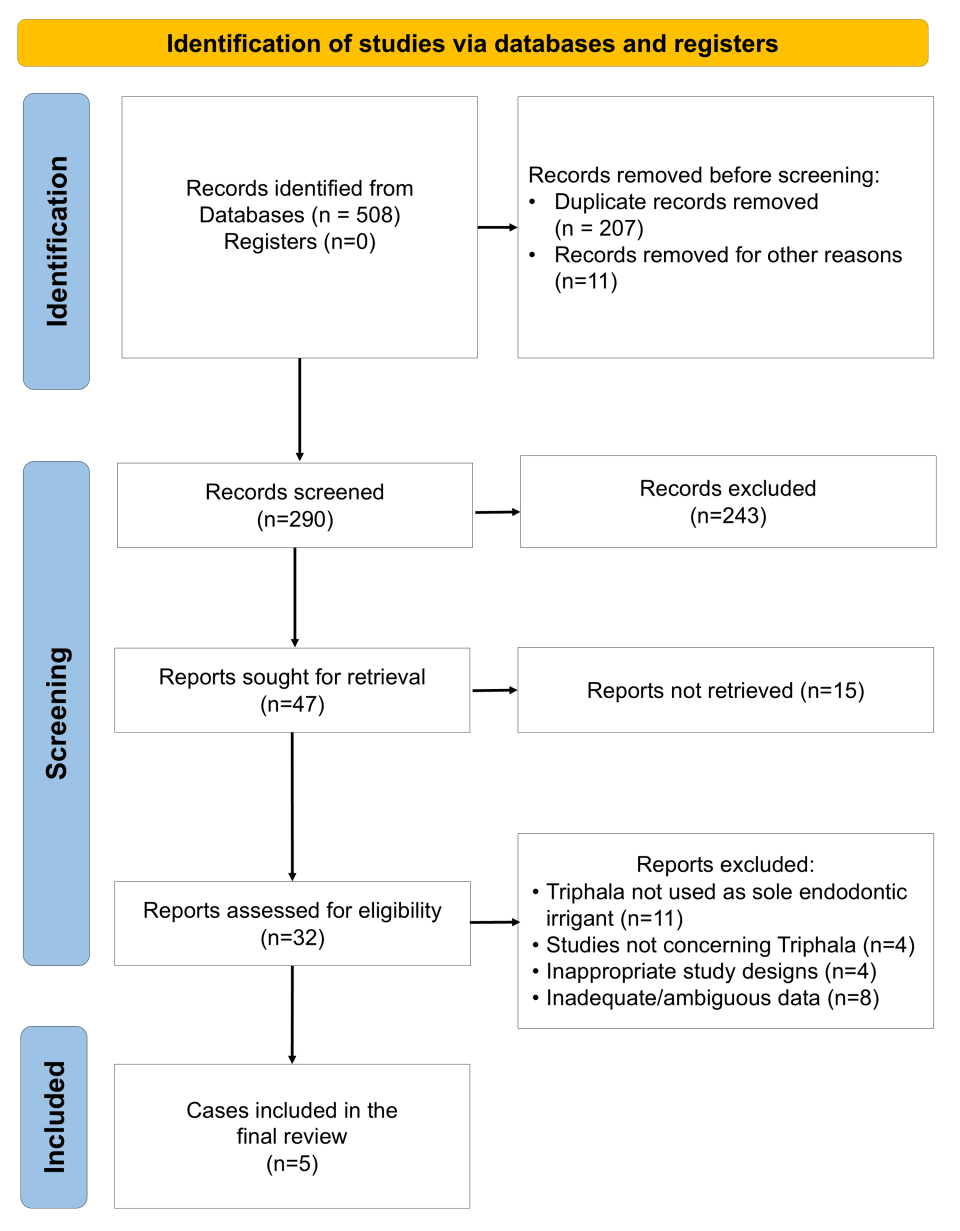
PRISMA flow diagram indicating the selection process of the articles in the present systematic review PRISMA: Preferred Reporting Items for Systematic Reviews and Meta-Analyses

A total of five studies were included in this systematic review following the application of eligibility criteria (Figure 1). Key study characteristics, including design, sample, Triphala formulation, comparator details, outcomes, and follow-up, obtained from these studies are summarized in Table 2.

**Table 2:** Characteristic data extracted from the studies included in the present systematic review CHX: chlorhexidine; CFU: colony-forming unit; DMSO: dimethyl sulfoxide; NaOCl: sodium hypochlorite; BHI: brain heart infusion; SD: standard deviation; RCT: randomized controlled trial; NS: not specified; N/A: not applicable

Author (Year)	Study design and setting	Sample (n; age range)	Tooth type	Triphala intervention (brand, formulation, concentration, protocol)	Comparator irrigant (type, concentration, protocol)	Key outcomes (quantitative where reported)	Follow-up
Thomas et al. (2017) [25]	In vitro microbiological study	n = 49; age not specified (extracted teeth)	Single-rooted primary teeth	Nilogram India Pvt. Ltd.; Triphala powder diluted 1:3 in 10% DMSO; used as irrigant with 5-min contact time	3% NaOCl; 5-min contact time	Mean CFU count against *Enterococcus faecalis*: Triphala 58.60 ± 16.63 vs. NaOCl 69.80 ± 19.57	Not applicable (in vitro)
Divya and Sujatha (2019) [26]	In vivo RCT (pediatric pulpectomy)	n = 30; 3-10 years; 19 males, 11 females	Maxillary and mandibular primary molars	IMPCOPS Ltd., Chennai; Triphala powder 5 mg/mL in 10% DMSO; 1 mL used as irrigant after instrumentation (contact time not specified)	Sterile saline; 1 mL after instrumentation (contact time not specified)	Post-irrigation microbial load (CFU/mL): Triphala 22.00 ± 18.11 vs. saline 13.46 ± 23.82; both groups showed significant reduction from baseline	Immediate post-irrigation only
Kiran et al. (2020) [27]	Ex vivo microbiological evaluation	n = 30; age not specified (infected extracted teeth)	Infected primary teeth (mixed types)	Triphala (brand not specified); 10% Triphala solution; 300 µL placed in agar wells for diffusion assay	0.5% NaOCl (Dakin’s solution); 300 µL in agar wells	Mean zone of inhibition against mixed microbial isolates: Triphala 23.83 ± 3.14 mm vs. NaOCl 20.97 ± 4.32 mm	24-hour incubation in a candle jar (ex vivo)
Bellal et al. (2024) [28]	In vitro microbiological study	n = 140; age not applicable (extracted teeth)	Single-rooted primary teeth	Triphala (brand not specified); solution prepared in 10% DMSO (exact concentration not stated); 5-min contact time in broth assays	2.5% NaOCl; 5-min contact time in broth assays	Growth/turbidity (mean ± SD): E. faecalis - Triphala 14.13 ± 1.25 vs. NaOCl 4.20 ± 1.01; *Candida albicans* - Triphala 13.60 ± 1.24 vs. NaOCl 3.53 ± 0.52	Post-incubation at 37°C for up to 96 hours
Pathivada et al. (2024) [29]	In vivo RCT (pediatric pulpectomy)	n = 150; 6-9 years; gender not reported	Multirooted primary molars	Morpheme Remedies (India); commercial Triphala juice (concentration not specified); 10 mL during instrumentation + 5 mL final rinse	2% CHX gluconate; 10 mL during instrumentation + 5 mL final rinse	Microbial reduction (CFU/mL): Triphala - blood agar 9.90 ± 11.56, anaerobic agar 6.50 ± 12.41; CHX - blood agar 3.80 ± 12.54, anaerobic agar 2.86 ± 13.01; both groups showed 100% clinical and radiographic success at all recalls	12 months (evaluations at three, six, nine, and 12 months)

Study Characteristics

All five studies were conducted in India; no eligible studies from other countries were identified despite the absence of country restrictions in the search strategy [25-29]. The evidence base comprised two in vivo RCTs on pediatric patients (Divya and Sujatha 2019; Pathivada et al. 2024), one ex vivo microbiological study using infected primary teeth (Kiran et al. 2020), and two in vitro experiments on extracted primary teeth (Thomas et al. 2017; Bellal et al. 2024) [25-29]. Thus, the dataset included two RCTs, one ex vivo study, and two in vitro studies.

Sample sizes ranged from 30 to 150, with a cumulative total of 399 teeth/participants across all studies. Age was reported in the RCTs only: Divya and Sujatha included children aged 3-10 years, and Pathivada et al. included children aged six to nine years [26,29]. Gender distribution was documented in one RCT (19 males, 11 females) [26]. Three studies evaluated primary molars (single- or multirooted), while two focused on single-rooted primary teeth [25-29].

Triphala was used as the test irrigant in all studies, but with variation in formulation and concentration. Most studies used Triphala powder dissolved in 10% dimethyl sulfoxide (DMSO), whereas one study used a 10% Triphala solution and another used a commercial Triphala juice preparation [25-29]. Irrigation/contact times were standardized at five minutes in the in vitro studies, while irrigation volumes (rather than precise exposure time) were reported in the clinical trials. Comparator irrigants included NaOCl at concentrations of 0.5%, 2.5%, and 3% (three studies), 2% CHX (one RCT), and sterile saline (one RCT) [25-29].

Microbial Outcomes

All five studies assessed antimicrobial efficacy as a primary outcome, using either quantitative CFU counts, turbidity measurements, or zone-of-inhibition assays against *E. faecalis*, *C. albicans*, or mixed microbial flora [25-29]. For comparisons with NaOCl, three studies (Thomas et al. 2017; Kiran et al. 2020; Bellal et al. 2024) evaluated Triphala versus 0.5-3% NaOCl [25,27,28]. In all three, NaOCl produced greater immediate antimicrobial reduction overall, whether measured as lower CFU counts or smaller residual growth/turbidity. However, Triphala consistently achieved significant reductions from baseline, demonstrating clear antimicrobial activity. In a zone-of-inhibition assay, 10% Triphala produced a larger mean inhibition zone than 0.5% NaOCl [27], although diffusion-based assays are sensitive to physicochemical properties and do not necessarily reflect clinical performance.

For comparisons with CHX and saline, two RCTs provided in vivo data. Divya and Sujatha (2019) compared Triphala with sterile saline and found that both irrigants, when used in conjunction with standard mechanical preparation, produced reductions in microbial load; the difference between groups was modest and not clearly clinically decisive [26]. Pathivada et al. (2024) compared Triphala with 2% CHX and reported lower post-irrigation CFU values in the CHX group on both blood agar and anaerobic media, but Triphala still produced substantial microbial reduction [29]. NaOCl was superior to Triphala in all three NaOCl-comparator studies (3/3), CHX showed lower CFU counts than Triphala in the single CHX-comparator RCT (1/1), and Triphala performed at least comparably to saline in the one saline-comparator RCT.

Clinical and Radiographic Outcomes

Only one study (Pathivada et al. 2024) included longitudinal clinical and radiographic follow-up [29]. In this RCT, children treated with either Triphala or 2% CHX were reviewed at three, six, nine, and 12 months. Both groups demonstrated complete clinical success (absence of pain, swelling, or sinus tract) and radiographic success (healing or stability of periapical status) throughout the 12-month period, with no reported treatment failures in any group. Thus, although CHX showed superior immediate microbial reduction, long-term clinical and radiographic outcomes were equivalent between Triphala and CHX over one year. No other included study reported long-term clinical or radiographic outcomes, as they were in vitro or ex vivo in design and focused solely on microbiological endpoints.

Safety and Adverse Events

Safety outcomes were explicitly reported only in the RCT by Pathivada et al., in which no adverse events, allergic reactions, or clinically apparent soft tissue irritations were observed in either the Triphala or CHX groups during the 12-month follow-up [29]. None of the other studies reported adverse events, which is expected for in vitro and ex vivo experiments. Overall, the available clinical evidence, though limited, suggests that Triphala is well tolerated when used as a root canal irrigant in pediatric patients.

RoB assessment

**Figure 2:**
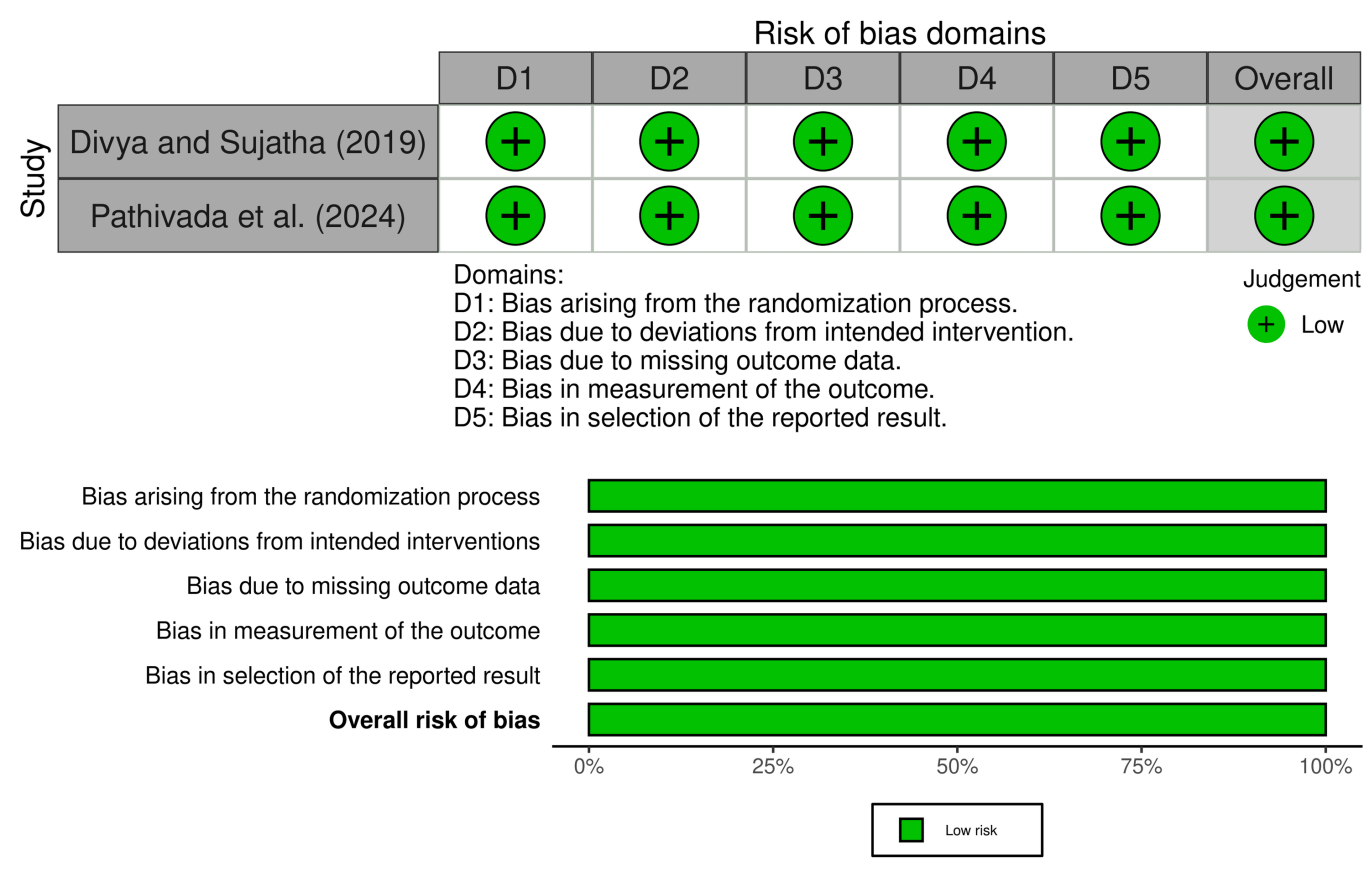
Risk of bias in the included randomized controlled trials using the Cochrane RoB-2 tool Cochrane RoB-2: Cochrane Risk of Bias 2.0 References [26,29]

Among the five included studies, two were RCTs and were evaluated using the RoB 2 tool. Both Divya and Sujatha (2019) and Pathivada et al. (2024) were judged to have a low overall RoB (Figure 2). These studies demonstrated proper randomization procedures, adequate handling of missing data, and valid outcome assessment protocols. Their adherence to methodological rigor enhances the reliability of their clinical findings and strengthens the evidence supporting the use of Triphala in vivo [26,29].

The remaining three studies - Thomas et al. (2017), Bellal et al. (2024), and Kiran et al. (2020) - were in vitro or ex vivo studies and were evaluated using the QUIN tool (Table 3). All three studies were rated as having a moderate RoB. While each study clearly stated its aims, provided detailed methodology, and used appropriate controls and statistical analysis, none of them justified their sample size or employed blinding in outcome assessment. Additionally, only two of the three studies incorporated randomization of samples. These methodological limitations, particularly the lack of blinding and sample size calculation, reduce the certainty and generalizability of their findings [25,27,28].

**Table 3:** Risk of bias in the included in-vitro/ex-vivo studies using the QUIN tool QUIN: Quality Assessment Tool for In Vitro Studies of Dental Materials

Study	Clearly stated aims	Sample size justification	Detailed methodology	Randomization of samples	Blinding of outcome assessment	Use of appropriate controls	Statistical analysis	Reproducibility (protocol clarity)	Overall risk of bias
Thomas et al. (2017) [25]	Yes	No	Yes	Yes	No	Yes	Yes	Yes	Moderate
Kiran et al. (2020) [27]	Yes	No	Yes	No	No	Yes	Yes	Yes	Moderate
Bellal et al. (2024) [28]	Yes	No	Yes	Yes	No	Yes	Yes	Yes	Moderate

Overall, the RoB assessment revealed that the evidence from the two RCTs is robust and credible. In contrast, the in vitro and ex vivo studies, although methodologically sound in many respects, show some limitations that must be considered when interpreting their results. These findings suggest that while preliminary laboratory evidence supports the antimicrobial efficacy of Triphala, stronger emphasis should be placed on the clinical trials when concluding its real-world application in pediatric endodontics.

Interpretation of the certainty of evidence

The GRADE approach evaluated the strength of the body of evidence across five key domains: RoB, inconsistency, indirectness, imprecision, and publication bias (not applicable here due to the limited number of studies) [34]. The evidence was categorized into four levels of certainty: high, moderate, low, or very low (Table 4).

**Table 4:** Assessment of certainty of evidence using the GRADE approach GRADE: Grading of Recommendations Assessment, Development and Evaluation; NaOCl: sodium hypochlorite; CHX: chlorhexidine; RCT: randomized controlled trial

Outcome	Number of studies	Study design	Risk of bias	Inconsistency	Indirectness	Imprecision	Certainty of evidence
Antimicrobial efficacy of Triphala compared to NaOCl	3	In vitro and in vivo	Moderate	Serious	Not serious	Serious	Low
Antimicrobial efficacy of Triphala compared to CHX	1	In vivo (RCT)	Low	Not serious	Not serious	Not serious	Moderate
Antimicrobial efficacy of Triphala compared to saline	1	In vivo (RCT)	Low	Not serious	Not serious	Not serious	Moderate

For the comparison between Triphala and NaOCl, data were extracted from three studies, including both in vitro and in vivo designs. The overall certainty of evidence for this outcome was graded as low. Although the findings consistently showed that Triphala had antimicrobial effects, there was a serious inconsistency due to variation in results across different experimental setups and formulations. Additionally, serious imprecision was noted due to variability in outcome measurements and a lack of standardized reporting. The moderate RoB in in vitro studies also contributed to downgrading the certainty.

In the comparison between Triphala and CHX, only one RCT was included. The study had a low RoB, and there was no serious inconsistency, indirectness, or imprecision in the outcomes. Therefore, the certainty of evidence was graded as moderate. This reflects a reasonable level of confidence in the findings, although further studies are needed to confirm and strengthen the results [29]. A single RCT also informed the comparison between Triphala and saline with low RoB, and no issues related to inconsistency, indirectness, or imprecision were identified. As a result, the certainty of evidence for this comparison was also graded as moderate [26].

Overall, the certainty of evidence was low for the comparison with NaOCl due to heterogeneity and limited clinical data. At the same time, it was moderate in comparison with CHX and saline, supported primarily by well-designed RCTs. These findings suggest a cautious but promising role for Triphala as a root canal irrigant, particularly when compared to less potent or safer alternatives in pediatric endodontics [25-29].

Discussion

The present systematic review evaluated the comparative effectiveness of Triphala, a traditional Ayurvedic polyherbal formulation, as a root canal irrigant in primary teeth. Across the five included studies, Triphala consistently demonstrated measurable antimicrobial activity against endodontic pathogens; however, the magnitude of its effect varied according to concentration, formulation, microbial species, and outcome assessment methods [25-29]. In direct comparisons, Triphala generally did not surpass conventional chemical irrigants such as NaOCl or CHX in terms of immediate microbial reduction, yet it reliably reduced microbial counts relative to baseline or inert comparators [25-29]. Taken together with its favorable biocompatibility profile and natural origin, these findings suggest that Triphala may be a promising adjunct or alternative irrigant in pediatric endodontics, particularly when safety concerns limit the use of higher-concentration synthetic irrigants.

Geographic and Methodological Heterogeneity

An important observation is that all included studies originated from India [25-29]. The literature search was not restricted by country; therefore, the exclusively Indian evidence base likely reflects both Triphala’s roots in Indian Ayurvedic practice, one of the world’s oldest traditional medical systems, still widely used in India today [35], and a true geographic concentration of research activity, but it may also indicate publication or language bias. Triphala, composed of the dried fruits of *T. bellirica, T. chebula, *and* E. officinalis*, is commonly used in Indian households as a health tonic, digestive aid, and oral rinse, and its components have been shown to possess antioxidant, antimicrobial, and immunomodulatory properties [36-38]. The consistent use of Triphala in Indian dental research reflects this cultural familiarity, yet also underscores the need for studies in diverse populations and international settings to assess its broader clinical applicability [37,38].

Methodologically, the body of evidence is heterogeneous: it comprises two in vivo RCTs, one ex vivo microbiological study, and two in vitro experiments on extracted primary teeth [25-29]. Sample sizes ranged from 30 to 150, tooth types and infection status varied, and follow-up was reported in only one trial [26,29]. Triphala was delivered as powder dissolved in 10% DMSO in most studies, whereas one trial used a commercial juice formulation, introducing further variability in active content and bioavailability [25-29]. Comparators spanned 0.5-3% NaOCl, 2% CHX, and sterile saline [25-29]. These differences in design, formulation, and outcomes were the primary reasons a quantitative meta-analysis was not attempted, and a narrative synthesis was adopted.

Efficacy of Triphala in the Context of Conventional Irrigants

When compared with NaOCl, three studies reported that NaOCl produced greater reductions in *E. faecalis* and *C. albicans *counts or turbidity, consistent with its well-known broad-spectrum antimicrobial and tissue-dissolving properties [25,27,28]. In our GRADE assessment, the certainty of evidence for Triphala versus NaOCl was rated as low, owing to the predominance of in vitro data, variability in protocols, and imprecision in effect estimates [25,27,28]. Nonetheless, Triphala still achieved significant microbial reductions in all NaOCl-comparator studies, indicating intrinsic antimicrobial potential [25,27,28]. Kiran et al. reported a larger mean zone of inhibition for 10% Triphala than for 0.5% NaOCl [27]; however, diffusion-based assays are highly sensitive to molecular size, solubility, and medium characteristics, and thus may not directly translate to clinical performance. In the RCT by Divya and Sujatha, Triphala and saline produced comparable microbial reductions when used alongside conventional mechanical debridement [26], suggesting that Triphala may perform at least as well as inert solutions when the mechanical component of treatment is adequate.

Of particular clinical relevance, Pathivada et al. observed that both Triphala and 2% CHX resulted in complete clinical and radiographic success at three, six, nine, and 12 months, with no reported adverse events [29]. Although this trial found CHX to yield numerically lower CFU counts than Triphala immediately after irrigation, the long-term outcomes were indistinguishable, supporting the notion that antimicrobial efficacy, host response, and healing dynamics all contribute to treatment success [29]. These findings mirror observations from adjacent endodontic literature, where calcium hydroxide pastes have been shown to be more effective than Ca(OH)_2_-impregnated points in eradicating *E. faecalis* from infected canals [15], and where flowable obturation systems such as GuttaFlow2 achieve filling quality and canal wall adaptation comparable to thermoplasticized gutta-percha in internal resorption defects [39]. Together, these data underline the need to balance antimicrobial strength with practicality, handling, and compatibility when integrating new agents such as Triphala into pediatric protocols.

Differences in antimicrobial efficacy among the included Triphala studies can plausibly be attributed to variability in concentration, preparation method, and inherent differences in microbial resistance. Triphala’s active phytoconstituents, including tannins, phenolic compounds, and flavonoids, vary in potency and relative proportion depending on the botanical source, processing, and extraction techniques [38,40]. The use of DMSO likely enhanced solubility and penetration of these components into dentinal tubules, whereas the commercial juice preparation employed in one trial may have exhibited reduced or less standardized active content [25-29,40]. Moreover, the antimicrobial activity of Triphala appears to be time-dependent in several models, with prolonged contact being necessary to disrupt established biofilms [36]. These factors, combined with differences in experimental design and endpoints, help explain the variability observed across studies and further justify the choice of narrative rather than quantitative synthesis.

Safety, Biocompatibility, and Clinical Acceptability

From a pediatric perspective, safety and biocompatibility are critical considerations, given the thin dentinal walls, open or resorbing apices, and proximity of primary roots to developing permanent tooth buds. High-concentration NaOCl, while highly effective antimicrobially, is associated with risks of tissue toxicity, chemical burns, and unpleasant taste, and its accidental extrusion beyond the apex can cause significant soft tissue damage [10,11]. In contrast, Triphala has been reported to exhibit relatively low cytotoxicity in experimental models and is widely consumed orally as a health supplement in Indian households [15-20,24,36]. In the available clinical trial included in this review, Triphala irrigation was not associated with any adverse clinical or radiographic events over 12 months [29]. Although formal biocompatibility testing in the context of irrigant extrusion and periapical tissues remains limited, the current evidence suggests that Triphala may offer a more favorable safety margin than some conventional irrigants, which is particularly relevant in young children and in teeth with open apices or advanced root resorption [10,11,36]. Furthermore, its herbal origin and familiarity in certain cultures may improve acceptance among parents seeking “natural” treatment options, provided that efficacy and safety are clearly explained.

Limitations

This review has several limitations. First, as noted above, all included studies were conducted in India [25-29], which restricts extrapolation to other populations and raises the possibility of geographic or publication bias despite the absence of country restrictions in the search strategy. Second, heterogeneity in study design (in vitro, ex vivo, in vivo), Triphala concentration and formulation, irrigation protocols, microbial targets, and outcome measures precluded formal meta-analysis and limited the certainty of pooled conclusions. Third, only one RCT provided longitudinal clinical and radiographic follow-up, and none reported patient-centred outcomes such as pain, treatment acceptability, or quality of life. Fourth, although we employed RoB 2, QUIN, and GRADE, several studies lacked sample size justifications, blinding, or detailed randomization procedures, contributing to the downgrading of certainty, particularly for comparisons with NaOCl. Finally, our conclusions are constrained by the small number of eligible studies and the reliance on surrogate microbiological outcomes, which may not fully reflect complex clinical healing processes.

Clinical Implications and Future Research

Within these limitations, the current evidence suggests that Triphala can achieve clinically meaningful microbial reduction in primary root canal systems and may be a reasonable alternative or adjunct in cases where the use of higher-concentration NaOCl is contraindicated or where a more biocompatible agent is desired. However, clinicians should recognize that NaOCl and CHX generally remain superior in terms of immediate antimicrobial effect, and Triphala should not be viewed as a direct replacement in all cases. Future research should prioritize well-designed, multicenter RCTs in diverse populations, using standardized Triphala formulations and concentrations, clearly defined irrigation protocols, and harmonized outcome measures including CFU reduction, clinical signs and symptoms, radiographic healing, and patient-reported outcomes. Studies that integrate Triphala-based irrigation regimens with established intracanal medicaments and contemporary obturation systems, including those with documented antimicrobial and sealing performance, would help clarify its role within comprehensive pediatric endodontic treatment. Longer-term follow-up and rigorous safety evaluations, particularly in relation to periapical tissues and developing permanent teeth, are essential before Triphala can be fully integrated into routine pediatric practice.

## Conclusions

The present systematic review highlights that Triphala exhibits promising antimicrobial efficacy as a root canal irrigant in primary teeth, demonstrating consistent microbial reduction across in vitro, ex vivo, and in vivo studies. While conventional irrigants such as NaOCl and CHX generally showed superior immediate antimicrobial action, Triphala’s favourable safety profile, biocompatibility, and natural origin make it a compelling alternative, particularly in pediatric endodontics where cytotoxicity and proximity to developing permanent teeth are of concern. Although limited primarily to Indian studies with methodological variability, the evidence suggests that Triphala can achieve clinically acceptable outcomes when used within a comprehensive treatment protocol. Future research through well-designed, multicentric RCTs with standardized formulations and long-term follow-up is essential to establish Triphala’s effectiveness and support its integration into routine pediatric dental practice.

## Appendices

**Table 5:** Detailed electronic search strategy across all databases

Database	Platform	Core search strategy (example, adapted to database syntax)	Filters/notes
PubMed	NCBI	(“Triphala”[Mesh] OR Triphala OR “tri phala” OR “tri-phala”) AND (“Tooth, Deciduous”[Mesh] OR “primary teeth” OR “primary tooth” OR “deciduous teeth” OR “primary molar*” OR “deciduous molar*”) AND (“root canal irrigant*” OR “root canal irrigation” OR “endodontic irrigant*” OR “endodontic irrigation”)	Standard PubMed field tags and MeSH terms used; reference lists of eligible articles also hand-searched.
MEDLINE	Ovid	(exp Triphala/ OR Triphala OR “tri phala” OR “tri-phala”) AND (exp Tooth, Deciduous/ OR (primary adj3 (tooth OR teeth OR molar$) OR deciduous tooth OR deciduous teeth)) AND (exp Root Canal Irrigants/ OR exp Root Canal Therapy/ OR “root canal irrigant$” OR “root canal irrigation” OR “endodontic irrigant$” OR “endodontic irrigation”)	Truncation symbols and adjacency operators adapted to Ovid syntax.
Embase	Elsevier	(‘triphala’/exp OR triphala OR ‘tri phala’ OR ‘tri-phala’) AND (‘deciduous tooth’/exp OR ‘primary tooth’ OR ‘primary teeth’ OR ‘deciduous teeth’ OR ‘primary molar*’) AND (‘root canal irrigation’/exp OR ‘root canal irrigant*’ OR ‘endodontic irrigant*’ OR ‘endodontic irrigation’)	EMTREE terms exploded where available; conference abstracts screened where relevant.
Scopus	Elsevier	TITLE-ABS-KEY(Triphala OR “tri phala” OR “tri-phala”) AND TITLE-ABS-KEY(“primary teeth” OR “primary tooth” OR “deciduous teeth” OR “deciduous tooth” OR “primary molar*” OR “deciduous molar*”) AND TITLE-ABS-KEY(“root canal irrigant*” OR “root canal irrigation” OR “endodontic irrigant*” OR “endodontic irrigation”)	Search limited to journal articles; duplicates removed during export and screening.
Cochrane Library (CENTRAL)	Wiley	(Triphala OR “tri phala” OR “tri-phala”) in Title/Abstract/Keywords AND (“primary teeth” OR “primary tooth” OR “deciduous teeth” OR “deciduous tooth”) AND (“root canal” OR endodontic)	CENTRAL trials register searched for randomized and quasi-randomized studies.
Google Scholar	Web	Iterative queries including: Triphala “primary teeth” “root canal irrigant”; Triphala “pediatric endodontics”; Triphala “root canal” antimicrobial. Approximately the first 200 records per query were screened by title/abstract.	Used to identify additional and grey literature; potentially relevant theses and non-indexed articles checked against eligibility criteria.
Global limits applied to all databases: Searches conducted from 1 January 2000 to 15 February 2025. No country restrictions were applied. Limited to English-language publications; human studies where applicable; duplicates removed at the screening stage.			
